# Professional Digital Counselling for Eating Disorders in Germany: Results of the DigiBEssst Project Survey on the Perspectives and Experiences of Health Professionals, Individuals With Eating Disorders, and Carers

**DOI:** 10.1002/erv.3164

**Published:** 2024-12-19

**Authors:** Anna Hofer, Sigrid Borse, Cäcilia Hasenöhrl, Kathrin Harrach, Stefan Ehrlich, Andreas Schnebel, Eva Wunderer

**Affiliations:** ^1^ Faculty of Social Work Landshut University of Applied Sciences Landshut Germany; ^2^ Faculty of Medicine, Technische Universität Dresden Dresden Germany; ^3^ Federal Association for Eating Disorders BFE Munich Germany; ^4^ Division of Psychological and Social Medicine and Developmental Neurosciences, Eating Disorders Research and Treatment Center at the Department of Child and Adolescent Psychiatry Faculty of Medicine Technische Universität Dresden University Hospital C.G. Carus Dresden Germany

**Keywords:** COVID‐19 pandemic, digitalisation in healthcare, eating disorders, online counselling, quality guidelines

## Abstract

**Objective:**

The research project DigiBEssst examines existing digital counselling services for individuals with eating disorders (ED) and carers in Germany. It highlights their experiences with digital counselling as well as those of the expert counsellors involved, aiming to derive quality criteria for digital counselling in ED.

**Method:**

A mixed‐methods design was adopted. Analysing the websites of 181 professional counselling centers, the research team identified 86 counselling centers for ED that offered online counselling. Initially, 29 centres participated in an online survey. Subsequently, in‐depth, semi‐structured interviews were conducted with professionals (*n* = 15), individuals with ED (*n* = 13), and carers (*n* = 10). The data were analysed using descriptive statistics and structured content analysis.

**Results:**

Less than half of the German counselling facilities offered online counselling. The participants emphasised the need for a specific concept, counsellors' profound expertise in ED and online counselling, sustainable funding, sufficient personnel and time resources, and secure platforms to ensure data protection and quality management. Access to professional services requires informative, user‐friendly websites, and social media presence.

**Conclusions:**

The identified prerequisites and quality criteria for professional online counselling developed in this project can provide recommendations for the conceptualisation of digital counselling services.

**Summary**

- Online counselling services offer low‐threshold access to professional support for people with eating disorders, but concrete measures, such as informative and user‐friendly websites, are crucial so that those seeking help can find professional counselling services online.
- Sustainable financial support is necessary for establishing digital counselling services.
- Professional digital counselling requires adherence to various quality criteria, including the professionals' qualifications.

## 1 Introduction

Eating disorders (ED) are severe psychosomatic illnesses for which prognosis improves with early intervention (Schmidt et al. 2016). Professional counselling centers are often the first point of contact for individuals with ED in their social environments. They constitute an indispensable element of the treatment chain (Federal Center for Health Education BZgA 2011).

During the COVID‐19 pandemic, ED symptoms and, consequently, the demand for accessible support increased, particularly among adolescent girls (Foroughi, Hay, and Mannan 2023; Haghshomar et al. 2022; Kohring et al. 2023). Concurrently, face‐to‐face counselling was often unavailable, prompting professional counselling centers to adopt new digital approaches (Strauß, Berger, and Rosendahl 2021; Weissman and Hay 2022; Wirkner et al. 2021). The high need for digital counselling could also stem from the high prevalence of ED among young people, for whom online activities are indispensable (de Zwaan 2015; Feierabend et al. 2023). Digital counselling can ensure comprehensive coverage, especially in areas traditionally underserved by counselling, such as rural regions (Wunderer et al. 2016a, 2016b).

Digital interventions have potential for individuals with ED. However, most studies have focused on internet‐based programs, including prevention and self‐help (Högdahl et al. 2023; Linardon et al. 2021; Pellegrini et al. 2022; Yim et al. 2021), and online therapies, including cognitive‐behavioural therapy (Dufour et al. 2022; Haderlein 2022; Melioli et al. 2016). Few have focused explicitly on digital counselling for ED (Schmidt‐Hantke et al. 2021). Therefore, the kind of online services that counselling centers offer for ED and how these are assessed by both professionals and those seeking help remain unclear.

Various associations have developed guidelines for providing online interventions; some, such as the German Association for Online Counselling DGOB (2020), specifically address digital counselling by offering recommendations on competencies for online counsellors and ethical‐professional guidelines, such as compliance with data protection regulations. Based on Waller et al. (2020), Taylor, Fitzsimmons‐Craft, and Graham (2020) created recommendations for online therapy for ED focussing on training, licencing, safety, privacy, and payment. Additionally, the professional literature includes occasional quality criteria, concepts, and recommendations by associations and counselling centers in specific areas (e.g., addiction help; Schaub et al. 2014). During the pandemic, specific guidelines and recommendations for digital counselling (e.g., Wenzel, Jaschke, and Engelhardt 2020), describing the methodological specifics of video and telephone counselling and providing recommendations for their application, were also issued. Despite these developments, there were no specific quality guidelines for online counselling for ED.

The 2‐year project ‘Digital counselling services of professional eating disorder counselling centers: Participatory inventory, evaluation, and development of quality guidelines (DigiBEssst),’ was funded by the German Federal Ministry of Health. It involved a collaboration between Landshut University of Applied Sciences and its Institute of Social Change and Cohesion Research IKON, and the Federal Association for Eating Disorders. The project DigiBEssst aimed to examine existing digital counselling services for individuals with ED and carers in Germany, highlighting their experiences of digital counselling as well as those of the counsellors involved. Based on these findings, the overarching goal of the study was to derive quality criteria for digital counselling in ED. The research questions were as follows.

1. Which digital counselling services are offered by professional counselling centers for ED in Germany?
2. What are the experiences of professionals, individuals with ED, and carers with digital counselling? What, from their perspectives, constitutes a professional digital counselling service, and what challenges do they face?

## 2 Methods

This project employed a mixed‐methods design and obtained data using an online questionnaire and semi‐structured interviews. The participants comprised professionals, individuals with ED, and carers. This project was approved by the Research Ethics Committee of the German Society for Social Work DGSA. The online survey was conducted entirely anonymously by an external entity with expertise in applied social research. The interviews were anonymised during transcription. Extensive study information and data protection statements were created for the participants, recruited nationwide through the Federal Association for Eating Disorders. The CROSS guidelines (Sharma et al. 2021) and COREQ checklist (Tong, Sainsbury, and Craig 2007) were considered in preparing this article and are provided as supplementary material.

### 2.1 Online Survey

An online questionnaire was developed to examine the digital services provided by professional counselling centers for individuals with ED as addressed in the first research question. It also included items relevant to the second research question, such as perspectives on and challenges in professional digital counselling. The questionnaire was used to collect information on counselling services, for example, access to online counselling; languages in which information and counselling is provided; media used in online counselling; purposes, target groups, settings of online counselling; and profession of online counsellors. Furthermore, the questionnaire addressed changes owing to the COVID‐19 pandemic and ED‐specific aspects of online counselling. The questions were developed by the research team based on the literature and feedback from ED counselling professionals from several counselling centers that were part of a ’practice kick off’ at the beginning of the project. The questionnaire also listed quality criteria for online counselling, derived from the literature. The respondents could evaluate these criteria in terms of their relevance and current implementation in their facility.

The questionnaire included open‐, semi‐open, and closed‐ended questions and rating scales. The item batteries assessed (1) standards related to institutional quality assurance (18 items, e.g., ‘Complaints management for online counselling clients’; endpoint‐verbalised rating scale addressing the relevance of standards: 1 = ‘not important at all’ to 5 = ‘very important’; endpoint‐verbalised rating scale addressing the implementation of standards: 1 = ‘very poor’ to 5 = ‘very good’); (2) qualifications in online counselling (e.g., ‘Knowledge of the legal frameworks for online counselling’; rating scales analogous to (1)); and (3) ED‐specific aspects of online counselling (e.g., ‘Potential physical endangerment may be less accurately recognised in text‐based online counselling compared with in‐person counselling’; endpoint‐verbalised rating scale: 1 = ‘does not apply at all’ to 5 = ‘applies completely’). Each item battery also provided an open field for additional comments. All closed‐ended and semi‐open questions were mandatory, ensuring no missing values.^1^

After a pilot test conducted in May 2022 with six online counsellors, five of whom specialised in ED, the online questionnaire was sent to ED facilities nationwide. Questionnaire administration was performed by a professional entity in applied social research using the SoSci Survey software. To identify professional ED facilities, a database of the Federal Center for Health Education BZgA and the Federal Association for ED BFE was used. The database adheres to strict criteria, including professional qualifications and compliance with recognised guidelines (e.g., German Society for Eating Disorders DGESS 2007; Herpertz et al. 2018). It includes both specialised and general counselling centers for ED and nutritional counselling. The research team thus identified 181 professional counselling centers for ED and examined whether they offered online counselling on their websites. The facilities' websites revealed that 86 (48%) offered online counselling. Therefore, responses were expected only from these counselling centers. Twenty‐nine facilities responded anonymously to minimise socially desirable answering; the survey management allowed only one response per facility. The survey period covered five weeks in May and June 2022. The response rate was 34% (29 out of 86), which is within the typical range for online surveys (Döring 2023, 407). The data were descriptively analysed using SPSS version 29.0 (IBM Corp., Armonk, NY, USA).

### 2.2 Semi‐Structured Interviews

Semi‐structured interviews were conducted to understand the perspectives and experiences of professionals (*n* = 15), individuals with ED (*n* = 13), and carers (*n* = 10) (Table 1), addressing the second research question. Professionals were recruited through a non‐probabilistic random sample from October to December 2022, mainly by the Federal Association for Eating Disorders, utilising its extensive network to include experts from various facilities across Germany. Interviews were held with professionals with expertise in online counselling; most of them specialised in ED. A professional with expertise in digital accessibility was also consulted to broaden the development of quality standards. From October 2022 to March 2023, individuals with ED and carers were recruited by asking ED facilities to launch a call for participation, for example, on social media. Interested individuals contacted the research team directly.

**TABLE 1:** Sample description of the participants in the interview study in the DigiBEssst project.

Professionals: 15	
Age	28–51 years, average age: 44 years; one did not disclose their age
Gender	Women: 12, men: 3
Location	Bavaria: 4, Saxony: 2, Hesse: 3, Hamburg: 2, Berlin: 2, Schleswig‐Holstein: 1, German‐speaking neighbouring countries: 1
Workplace	Large cities (population ≥ 100,000).
Professional backgrounds	Social workers/Social educators: 5, educators: 3, psychologists: 2, nutrition psychology: 1, adult education: 1, communication: 2, theatre studies: 1
Professional experience in ED	*n* = 11; ranging from 2 to 5 years: 5; over 15 years: 6
Online counselling experience	*n* = 14; from 2 years to over 20 years
Online counselling modalities	Text: 1, video: 5, video and text: 5
Certification in online counselling	Certified training in online counselling recognised by the German Association for Online counselling DGOB: 2
Individuals with ED: 13	
Age	21–49 years; average age: 31 years (7 not older than 25 years)
Gender	Women: 11, men: 2
Location	Bavaria: 6, Hesse: 3, Berlin: 1, Baden‐Württemberg: 1, Rhineland‐palatinate: 1, North Rhine‐Westphalia: 1
Type of ED (as indicated by respondents)	Anorexia nervosa: 6, Bulimia nervosa: 2, Binge ED: 2, mixed (anorexia and bulimia): 1, atypical: 2
Carers: 10	
Age	45–60 years; average age: 52 years
Gender and parental role	Women/mothers: 8, men/fathers: 2
Location	Bavaria: 2, Baden‐Württemberg: 1, Berlin: 1, Brandenburg: 2, Hamburg: 1, Hesse: 3

Individuals with ED and carers had used online counselling services for ED at least once. In no case were individuals with ED and their family members interviewed. The interviews were conducted from October 2022 to February 2023 by two female project staff members (MA in Clinical Social Work and doctoral candidate and BA in Social Work pursuing an MA in Clinical Social Work) mainly using Zoom. The interviewers rigorously prepared by reviewing and internalising extensive literature on semi‐structured interviews (Helfferich 2022; Misoch 2019) and conducted a pretest interview with an online counselling professional for ED to refine the interview guide and techniques. During the information phase, the interviewers introduced themselves and explained their research interests. Participants received written information before the interview, and any questions could be clarified in advance. The interviews were audio‐recorded and postscripts were written. The average duration of interviews with professionals, individuals with ED, and carers was 66 min, 57 min, and 71 min, respectively. To the interviewers' knowledge, only the participants and researchers were present during the interviews.^2^

A professional transcription service was commissioned to process the interviews, and the transcripts were not returned to the interviewees. The data were analysed using qualitative structured content analysis according to Kuckartz and Rädiker (2022) and the software MAXQDA in seven steps; the two interviewers also performed the coding. In step 1, text work was conducted by highlighting important passages in the text, followed by developing main thematic categories according to the research questions (step 2), and an initial coding process in which text sections of the entire material were assigned to main categories (step 3). All text sections in each main category were considered collectively to inductively develop sub‐categories (step 4) and assign the material to them (step 5). The categories in the three category systems^3^ vary slightly depending on the interview group, based on the topics explored in the interviews using the interview guide and the aspects brought up by the interviewees. For example, individuals with ED and carers provided more detailed answers than professionals regarding how they perceive the findability and design of access to online counselling. After structuring and condensing the material, the specificities, commonalities, and differences were summarised and analysed (step 6) and presented in a results report (step 7). This approach was used to categorise the data thematically into areas such as ‘Access and Setting,’ ‘Attitude and Self‐Care,’ and ‘Counselling Competencies and Challenging Situations,’ leading to the identification of good practices and prerequisites for online counselling in ED, and therefore forming the basis for the development of quality guidelines.

## 3 Results

### 3.1 Online Survey of Professional Counselling Centers

#### 3.1.1 Online Counselling Services for ED

Forty‐one percent of the counselling centers participating in the online survey (*n* = 29) introduced digital counselling only owing to the pandemic. Seventy‐two percent of the participating counselling facilities provided access to counselling through their websites, 52% (also) through a specific counselling portal, and 24% (also) through social media. At the time of the online survey, most counselling centers offered online counselling through email (83%) and/or video (79%). Chat‐based counselling was used by 31% of the participants, and messenger‐based counselling by 10%. Some institutions expressed an intention to use these media in the future (48% for chats and 17% for messengers). None of the participating counselling centers provided online counselling through a forum. Blended counselling, a combination of online and in‐person sessions, was practiced by 83% of the centers. Individual counselling for those with ED was reported as the most frequent setting for digital counselling (97%), followed by counselling for carers (76%). Approximately 62% of counselling centers offered digital counselling in English, and 7% could provide it in sign language. Online counselling in other languages was not offered by the included centers.

#### 3.1.2 Prerequisites and Conditions for Professional Online Counselling for ED

Only one counselling center created its own concept for online counselling. Most centers adapted in‐person counselling concepts for online use (62%) and/or followed general standards for online counselling (21%) or operated without a specific concept (24%). Overall, 48% considered a specific concept for online counselling important, that is, they chose four or five on the endpoint‐verbalised rating scale (not important at all [1] to very important [5]; *M* = 3.45, SD = 0.95). Regarding the criteria for institutional quality assurance, technical equipment was rated as the most important, but not the best implemented (*M* = 3.45, SD = 1.15). Online counselling evaluation, complaints management for those seeking online counselling, and financial support for online counselling were rated as poorly implemented (*M* ≤ 2.5) and moderately relevant (*M* > 3.5). The 11 items assessing the professionals' qualifications for online counselling for ED were rated higher in relevance than in their implementation in counselling facilities. Regarding the other competencies assessed, the professionals rated ‘Consideration of self‐care in online counselling’ as the most relevant and best implemented. Knowledge of the legal frameworks and competence for crisis intervention and emergency planning were considered poorly implemented but highly relevant (Table 2).

**TABLE 2:** Assessment of the relevance and implementation of aspects regarding institutional quality assurance of online counselling and the specific competencies and knowledge of counsellors for online counselling in the online questionnaire of the DigiBEssst Project, May/June 2022.

	How relevant is this aspect?	How is this aspect implemented in your counselling center?
Institutional quality assurance for online counselling		
Technical equipment for online counselling	*M* = 4.72 (SD = 0.53, *n* = 29)	*M* = 3.45 (SD = 1.15, *n* = 29)
System stability in online counselling	*M* = 4.62 (SD = 0.62, *n* = 29)	*M* = 3.17 (SD = 0.97, *n* = 29)
Defined timeframe for expecting responses in online counselling	*M* = 4.55 (SD = 0.69, *n* = 29)	*M* = 4.21 (SD = 1.07, *n* = 28)
Defined procedures in crisis situations in online counselling	*M* = 4.52 (SD = 0.87, *n* = 29)	*M* = 3.43 (SD = 1.30, *n* = 28)
Networking and referral of online counselling clients to appropriate facilities	*M* = 4.38 (SD = 0.78, *n* = 29)	*M* = 4.21 (SD = 0.90, *n* = 29)
Coordination of organisational processes regarding online counselling	*M* = 4.34 (SD = 0.61, *n* = 29)	*M* = 3.75 (SD = 1.14, *n* = 28)
Staff resources for online counselling, administration and technical support for online counselling	*M* = 4.14 (SD = 0.99, *n* = 29)	*M* = 2.97 (SD = 1.05, *n* = 29)
Regulations for documentation of online counselling (e.g., storage of online counselling session data)	*M* = 4.03 (SD = 0.98, *n* = 29)	*M* = 3.75 (SD = 1.14, *n* = 28)
Financial support for online counselling	*M* = 3.97 (SD = 1.05, *n* = 29)	*M* = 2.56 (SD = 1.12, *n* = 27)
Specific qualifications for professionals providing online counselling in the counselling center (e.g., further training)	*M* = 3.93 (SD = 1.13, *n* = 29)	*M* = 3.15 (SD = 1.38, *n* = 26)
Home office with adequate equipment for online counselling	*M* = 3.83 (SD = 1.26, *n* = 29)	*M* = 2.90 (SD = 1.47, *n* = 29)
Multi‐professionalism in online counselling (involvement of different professions, depending on the specific needs of the clients)	*M* = 3.83 (SD = 1.04, *n* = 29)	*M* = 3.27 (SD = 1.25, *n* = 26)
Evaluation of online counselling (e.g., satisfaction of clients)	*M* = 3.79 (SD = 0.77, *n* = 29)	*M* = 2.50 (SD = 1.23, *n* = 28)
Defined time budget for online counselling (e.g., time per client, determination of the number of contacts)	*M* = 3.55 (SD = 1.30, *n* = 29)	*M* = 3.52 (SD = 1.19, *n* = 27)
Complaints management for online counselling clients	*M* = 3.55 (SD = 1.02, *n* = 29)	*M* = 2.43 (SD = 1.03, *n* = 28)
Specific concept for online counselling	*M* = 3.45 (SD = 0.95, *n* = 29)	*M* = 2.96 (SD = 0.81, *n* = 27)
Intervision, team meetings, case discussions specific to online counselling	*M* = 3.41 (SD = 1.18, *n* = 29)	*M* = 3.00 (SD = 1.12, *n* = 25)
External supervision specific to online counselling	*M* = 3.07 (SD = 1.25, *n* = 29)	*M* = 2.54 (SD = 1.30, *n* = 26)
Competencies and knowledge of counsellors for online counselling		
Consideration of self‐care in online counselling (e.g., setting boundaries)	*M* = 4.62 (SD = 0.49, *n* = 29)	*M* = 4.04 (SD = 0.79, *n* = 28)
Crisis intervention and emergency planning in online counselling	*M* = 4.59 (SD = 0.57, *n* = 29)	*M* = 3.59 (SD = 1.08, *n* = 27)
Assessment of indication and contraindication of online counselling for people with eating disorders	*M* = 4.55 (SD = 0.57, *n* = 29)	*M* = 3.86 (SD = 0.89, *n* = 28)
Knowledge of legal frameworks for online counselling	*M* = 4.52 (SD = 0.74, *n* = 29)	*M* = 3.39 (SD = 1.13, *n* = 28)
Establishment of professional working relationships in online counselling (e.g., facilitating a helping process despite anonymity, handling lower commitment in online counselling)	*M* = 4.48 (SD = 0.63, *n* = 29)	*M* = 4.00 (SD = 1.02, *n* = 28)
Technical competence for online counselling (e.g., computer skills)	*M* = 4.41 (SD = 0.73, *n* = 29)	*M* = 4.04 (SD = 0.98, *n* = 27)
Specific reading, writing, and text competence for online counselling (e.g., reading between the lines and responding, using emoticons and abbreviations, fast typing)	*M* = 4.34 (SD = 0.77, *n* = 29)	*M* = 3.73 (SD = 1.19, *n* = 26)
Using different forms of online counselling with their respective special features appropriately (e.g., email, video, chat)	*M* = 4.34 (SD = 0.72, *n* = 29)	*M* = 3.74 (SD = 1.10, *n* = 27)
Establishing a reflective open attitude towards online counselling	*M* = 4.31 (SD = 0.71, *n* = 29)	*M* = 4.04 (SD = 0.96, *n* = 28)
Dealing with difficult communication situations in online counselling (e.g., prolific writers, communication breakdowns)	*M* = 4.28 (SD = 0.65, *n* = 29)	*M* = 3.68 (SD = 1.02, *n* = 28)
Specific methodological competence for online counselling (e.g., using tools such as online whiteboard, adapting methods to online counselling)	*M* = 3.93 (SD = 0.59, *n* = 29)	*M* = 3.11 (SD = 1.09, *n* = 27)

*Note:* Endpoint‐verbalised rating scale: 1 = not important at all (relevance) or very poor (implementation) to 5 = very important (relevance) or very good (implementation).

#### 3.1.3 Specifics of Online Counselling for Individuals With ED

**FIGURE 1:**
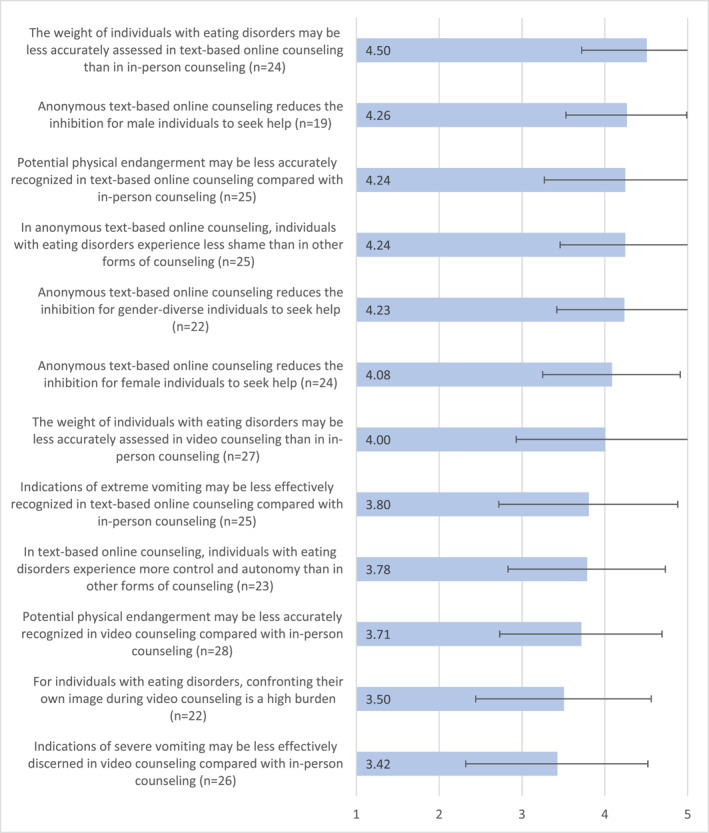
Mean values regarding the assessment of eating disorder‐specific aspects of online counselling in the online questionnaire of the DigiBEssst project, May/June 2022, endpoint‐verbalised rating scale: 1 = does not apply at all to 5 = applies completely.

Figure 1 illustrates, based on the mean values, the extent to which the professionals agreed with the questionnaire statements regarding ED‐specific aspects of online counselling. Professionals largely agreed with the ED‐specific statements (*M* ≥ 3.5), although some variation was apparent.

### 3.2 Key Interview Findings

The three topics hereafter emerged as key concerns across all three interview groups.

#### 3.2.1 Access to Online Counselling

The interviews with professionals, individuals with ED, and carers revealed that online counselling can be a low‐threshold, location‐independent, and time‐flexible option to reach people seeking help for ED:

“The threshold to even accept help and recognise that this is an illness is very, very high, especially with ED […]. And I find these online services very, very helpful because they provide an initial alternative way to have the courage to ask, ‘do I really have an illness or what can I do?’ And because it's so anonymous at first and very low‐threshold. So, I think it's important to expand that. […] Especially with ED, I have experienced firsthand […] how hard it is to seek help” (individual with ED 1, para. 17).

However, individuals with ED and carers reported that finding online professional counselling services can be challenging. Particularly, social media also carries the risk of introducing unprofessional services. Individuals, carers, and professionals desired low‐threshold access to professional online counselling, which requires a clear, user‐friendly, and informative website and, according to professionals and individuals with ED, a presence on social media. All three groups advocated for access through the ‘messenger’ medium, but professionals also expressed difficulties as currently, no satisfactory messenger service combines high target‐group reach with data protection compliance.

All three groups also agreed that digital counselling for ED is relevant for people of all ages, providing low‐threshold support for those who prefer counselling in familiar environments or find in‐person counselling difficult to access: ‘*[…], I have to say, it's obviously much easier to fit it into your day when you don't have to drive anywhere*’ *(carer 3, para. 47).* Counselling centers should consider diverse target groups, including people of all ages, genders, cultural backgrounds, and social situations, as well as those with physical and cognitive impairments or burdens. Efforts should be made to address these groups specifically through digital services, including providing explicit information about the target audience(s) on the website. Additionally, the interviewees pleaded for the participation of help seekers in selecting the counselling format. According to professionals and individuals with ED, blended counselling can be a good way to provide everyday support.

#### 3.2.2 Resources for Online Counselling

The interviewed professionals emphasised the importance of a well‐defined concept for online counselling, ideally developed before the service is launched: ‘*That means, I always say, first the idea, then the computer*’ (*professional 6, para. 25*). This also includes sustainable financial planning. Professionals mentioned obstacles such as time‐limited project funding for online counselling and regionally localised funding structures:

“Yes, I think overall that the funding opportunities for online counselling in the area of ED are very patchy. It needs more funding. It's also unfortunate that we rely on private foundation grants and don't get more support from the government. This is such important work being done. Our foundation funding runs out […], and it's incredibly difficult to secure new grants. We know we want to continue our work because the need is so high, but that brings challenges and new funding concepts. It shouldn't be like this […] I would rather invest this time in online counselling than in securing funding” (professional 12, para. 94).

Consequently, some of these services are partially fee‐based, which can create an additional hurdle for seeking support as described by all three interview groups:

“Yes, I think it would definitely be helpful if it could be free, because having to spend money is an additional barrier. Especially when you’re younger and don’t want to ask your parents for money, but instead have to come up with it yourself, it becomes even more difficult […]” (individual with ED 7, para. 89).

Transparently communicating costs and payment methods is crucial to avoid misunderstandings. Professionals emphasised the need for more personnel and time resources, as well as a user‐friendly counselling platform that meets data protection requirements and ensures data security for clients. In order to reach diverse target groups, the provision of online‐based counselling should not lead to the discontinuation of other counselling formats owing to a lack of resources. Professionals also pointed out that text‐based counselling often takes more time than face‐to‐face or video counselling owing to multiple steps in the process of reading and writing; this should be considered regarding time allocation and team coordination. Resources must also be allocated for quality management, regarding *‘structural, process, and outcome quality’ (professional 9, para. 69)*. According to the interviewees with ED, this can be effectively achieved through anonymised online surveys or direct feedback during counselling sessions, once a good relationship has been established.

#### 3.2.3 Competencies for Online Counselling

Professionals, individuals, and carers stated that online counsellors need professional counselling knowledge and competencies as well as technical and ED‐specific expertise acquired through their studies and professional experience:

“Very helpful in the online counsellors was their calmness, confidence, and expertise on the topics. […] I believe that the combination of sensitivity, expertise, and learned techniques made me leave the session thinking, yes, it wasn’t just a garbage can where I told all the terrible things about how I feel, but it truly helped” (carer 2, para. 69).

All three groups also supported (certified) additional training for professionals. According to professionals, specific knowledge in text‐ and video‐based counselling, such as specific reading and writing techniques (e.g., the four‐slide concept by Knatz 2009), is desirable to compensate for limited or missing nonverbal and paraverbal signals and to build a relationship. ‘*The relationship is also the key element and the main factor of effectiveness in online counselling*’ *(professional 6, para. 61)* and, with specific reading and technical writing skills, can be successfully established in digital counselling formats:

“Yes, I think that having a strong sense of language or being highly attentive to written language can be very helpful in text‐based online counselling. Also, allowing yourself to ask: what images come to mind when I read this text? And then: what does it trigger in me? […] And continually bringing that into the conversation. For example, ‘I have an idea about this, what do you think?’ […] This way, what’s missing—like facial expressions, gestures, and all the atmospheric cues—can be somewhat compensated for in online counselling” (professional 12, para. 82).

Seeing one's own image on the screen is a challenging situation that needs to be addressed specifically in video counselling: ‘*Video counselling would be extremely difficult for me. I never wanted that. I had the opportunity to use it, but I don't like it. It's always a huge effort for me to participate in online meetings because I see myself the entire time*’ *(individual with ED 1, para. 27).* One professional called it the ‘*enemy in the room*’ *(professional 5, para. 35)* for individuals with ED, and interviewees pointed out that seeing one's own image can also be distracting for professionals. Individuals with ED find it helpful when they can minimise their own image or turn off the camera and when professionals openly address the issue during the counselling session.

The interviewees also emphasised the need to reflect on the possibilities and limitations of digital counselling, especially in crisis situations (e.g., suicidality). On the one hand, online counselling offers the opportunity for anonymity in crises, which can make it easier for clients to open up. Additionally, it can allow for more immediate support. Therefore, the three interviewee groups agreed that there are no universal exclusion criteria for online counselling for ED; suitability varies depending on the individual and the specific situation. On the other hand, clients might have to be quickly referred to an inpatient facility owing to their physical condition (e.g., very low body weight) or mental distress. Therefore, transparency regarding the limits of the online counselling service is essential for clients as well as professionals:

“For example, if I’m offering email counselling, it’s essential to communicate clearly and transparently that we are not an acute crisis intervention service, as I can’t respond immediately. It’s also crucial that facilities have a crisis intervention guide, which they usually have for face‐to‐face counselling. But it’s important to ensure that this is also part of the concept for online formats” (professional 6, para. 49).

## 4 Discussion

This study aimed to explore existing digital counselling services for individuals with ED and carers in Germany, focussing on their experiences with digital counselling, as well as the perspectives of the counsellors providing these services. The overarching goal was to use these insights to develop quality criteria for digital counselling in ED. An online questionnaire and semi‐structured interviews were used. The COVID‐19 pandemic and its associated measures have prompted counselling centers to introduce new digital services or focus more on them (Strauß, Berger, and Rosendahl 2021; Weissman and Hay 2022; Wirkner et al. 2021; s. online survey^4^). However, according to their websites, only half of the counselling centers offer online counselling. This aligns with the study by Lincke et al. (2022) that revealed the slow integration of e‐mental health and home treatment into the German healthcare system; however, these approaches seem to be effective.

The interviews with professionals, individuals with ED, and carers indicate that online counselling is an effective way to reach individuals seeking help for ED. The anonymity and autonomy offered by digital counselling formats support the self‐determination of clients, allowing them to decide when and to what extent they want to open up (s. interviews; Hintenberger 2021, 164). This lowers the barrier for initial contact, which is often higher for individuals with ED owing to stigma and feelings of shame (s. also online survey; Haderlein 2022, 181). In light of the study by Daugelat et al. (2023, 752), which identifies stigma, shame, and guilt as key obstacles to seeking treatment, it becomes clear how crucial digital counselling formats can be. To give individuals the opportunity to receive professional support services according to their preferences and needs, a diverse range of options with multiple access points is needed, including different languages, age groups, and genders (s. interviews). Messenger services are widely used by young people (Shell youth study 2024, 21), can provide low‐threshold access to online counselling (Engelhardt and Piekorz 2022), and were considered a target‐oriented medium for online counselling by the interview participants. However, this medium is offered by only a few counselling centers, possibly because of the difficulty in complying with legal requirements (s. interviews and online survey).

Professional online counselling requires adequate financial resources (Burghardt and Lehmann 2022, 19) to ensure sufficient personnel and time resources, which significantly impacts whether standardised quality criteria for digital counselling services can be adhered to, including the qualification and training of staff, data protection‐compliant technical equipment, and continuous quality assurance (s. interviews and online survey). Increased financial resources are necessary, particularly as online counselling could help save costs in the healthcare sector by intervening early, therefore improving the prognosis of ED (Schmidt et al. 2016).

Furthermore, counsellors' professional qualification, along with continuous training and further education, is crucial (s. interviews; DGOB, 2020). The fact that the interviewed professionals, individuals with ED, and carers emphasised successful working relationships specifically in online counselling suggests that findings from online therapy—indicating that positive working relationships are possible online (Sucala et al. 2012)—could also be transferable to online counselling.

However, it should be noted that digital counselling alone can often not provide sufficient support for individuals with ED, and online counsellors act as gatekeepers drawing on strong and broad professional networks (s. interviews). The physical distance requires particularly attentive practices, for example, in assessing the physical condition of individuals with ED (s. interviews).

### 4.1 Strengths and Limitations

This study, to the authors' knowledge, is the first to systematically capture digital counselling services for ED in Germany, and evaluate the experiences of professionals, individuals with ED, and carers with these services.

Twenty‐nine questionnaires could be included in the analysis, which is relatively low for a nationwide survey, despite the response rate of 34% being within the normal range for online surveys (Döring 2023, 407). The online questionnaire survey and interviews exclusively involved professionals, individuals with ED, and carers who had already experienced digital counselling. Therefore, those with an existing interest in digital counselling formats were more likely to participate in the survey.

The sample of 15 professionals, 13 individuals with ED, and 10 carers represents a broad spectrum of experiential knowledge across different age groups and genders (although all participants identified as men or women). The sample included different professional backgrounds, various types of ED, and different family roles (although only mothers and fathers). Additionally, experiences with both video‐ and text‐based counselling in various settings, such as individual and group counselling, were captured. The number of participants is considered sufficient, as a degree of data saturation was reached during the analysis; with each additional interview, only a few new aspects emerged in the evaluation. However, no adolescents were included, resulting in a lack of perspective from this age group. The effects of respondent and interviewer bias also cannot be entirely eliminated.

As part of the project DigiBEssst, quality guidelines for professional digital counselling have been developed specifically for ED (Hofer et al. 2023) by linking and critically examining preexisting literature and empirical findings. These guidelines can serve as a basis for adapting concepts for online counselling, therefore closing a gap in research and practice. Future research could explore the usefulness of the guidelines for professionals, individuals with ED, and carers.

### 4.2 Practical Implications

The quality guidelines derived from the results include recommendations regarding access to online counselling for ED (Hofer et al. 2023, 20–29). This encompasses aspects such as the design of and information on the websites, client participation in the choice of the counselling format, and conceptual design of messenger counselling as well as blended counselling. Further, the guidelines emphasise the importance of financial resources, recommending early and long‐term financial planning and advocating for political acceptance by highlighting the potential of online counselling for ED (Hofer et al. 2023, 84–88). They also address the need for adequate personnel and time resources, such as optimised staff planning, allocating sufficient time resources, and defining fixed working hours for online counselling (Hofer et al. 2023, 90–92). Additionally, technical and legal considerations are covered, including the selection of a secure online counselling platform that meets data protection requirements, as well as quality management focussing on the three classic dimensions: structural, process, and outcome quality (Hofer et al. 2023, 94–113). Another chapter of the quality guidelines (Hofer et al. 2023, 46–52) is dedicated to the qualifications and competencies of online counsellors, emphasising foundational professional counselling knowledge and skills acquired through education and work experience. Specific skills concerning ED and family support, along with additional training and continuous professional development for online counselling, are also important. Relationship‐building is highlighted, with suggestions for fostering relationships by providing a sense of security (Hofer et al. 2023, 54–58). Strategies for managing challenging situations and limitations, such as handling severe ED symptoms and comorbidities, and the self‐view challenges during video counselling, are outlined, as well as guidance on networking and further development (Hofer et al. 2023, 60–79). The guidelines were developed based on the existing literature and the data collected. However, future studies are needed to explore the specific elements of online counselling that were identified in this study and clearly outlined in the guidelines, along with their effects. Table 3 presents the main components of the quality criteria of the key findings discussed in this article.

**TABLE 3:** Key components of the quality criteria.

**Access to online counselling**
Design and information on the website as well as public relations for low‐threshold accessibility (20 ff.) Participation of clients in the choice of counselling format, setting, scheduling, and selection of the counsellor (23 f.) Focus on diverse target groups in terms of age, gender, culture, and language, individuals with disabilities, and people in various social situations, with different types of eating disorders and roles as relatives (24 ff.) Counselling via messenger (26 ff.) Blended (online) counselling (28 f.) Multi‐person settings (30 ff.)
**Resources for online counselling**
**Concept**: Written and publicly accessible concept for online counselling, including client participation and continuous updates (82 f.) **Financial aspects**: Early and long‐term financial planning (84 f.); highlighting the potential of online counselling for eating disorders to gain political acceptance (85 f.); establishing free (or partially free) online counselling services (87); transparency regarding costs and payment methods (87 f.) **Personnel and time resources**: Adjusting personnel planning and budgeting resources (90 f.); allocating time resources and defining fixed working hours (91 f.); communicating response times to clients (92) **Collaboration within the team and with external partners**: Coordination of organisational processes and documentation (72 f.); interdisciplinarity (73); intervision, team meetings, and case discussions (74); awareness of the possibilities and limitations of online counselling for eating disorders and educating clients (77); broad networking; (77 ff.); supervision (79) **Technology:** Professional, up‐to‐date technical equipment and selection of a data‐protection‐compliant online counselling platform (95 ff.) **Legal frameworks:** Basic legal knowledge and keeping up‐to‐date with legal regulations (101); collaboration with legal and IT professionals and adherence to current legal standards (101); handling data and documentation (102 ff.); confidentiality, transparency, and consent (105 ff.); ensuring privacy (107); special considerations for minors (108 f.) **Quality management:** Continuous quality assurance from the outset and openness to change (110 ff.); orientation towards the three classic dimensions of structure, process, and outcome quality (111 ff.)
**Competencies for online counselling**
**Qualifications and competencies of online counsellors:** Professional counselling knowledge and skills from education and professional experience, along with specific skills for counselling for eating disorders (46 f.); additional training and continuous professional development for online counselling (48); technical and media competencies (48 f., 97 ff.); specific competencies for text‐based counselling (49 f.); specific competencies for video‐based counselling (51 f.) **Relationship building:** General relationship‐building in online counselling (54 f.); fostering a sense of security in relationships (55 f.); relationship‐building in text‐based counselling (56 ff.); relationship‐building in blended counselling (58) **Managing difficult situations and boundaries:** Severity of eating disorder symptoms and comorbidities (62); written communication and frequent or excessive messaging (63); unreliable enquiries (63); stagnating counselling processes and contact terminations (63 f.); counselling in the client's home (64); crisis situations (64 ff.); challenges of online counselling (e.g., confronting one's own image in video counselling) (122 ff.)

## 5 Conclusion

In summary, online counselling emerged as a flexible and low‐threshold option, especially valued for its anonymity and accessibility, though finding reliable services can be challenging, particularly on social media. While email and video counselling were the most common formats, several centers showed interest in adopting chat and messenger‐based counselling in the future, alongside blended approaches. The interviewees emphasised the need for sustainable funding, adequate resources, and training in digital counselling techniques, as well as clear communication of expectations and service limitations, e.g. in crisis situations. It is noteworthy that many aspects of digital counselling, particularly in the context of ED, remain insufficiently substantiated by scientific studies, highlighting the need for further research in this area.

## Ethics Statement

The study was approved by the Research Ethics Committee of the German Society for Social Work DGSA.

## Conflicts of Interest

The authors declare no conflicts of interest.

## Supporting information

Supporting Information S1

Supporting Information S2

Supporting Information S3

Supporting Information S4

Supporting Information S5

Supporting Information S6

Supporting Information S7

## Data Availability

The datasets of the current study are not publicly available due to privacy and ethical restrictions but are available from the corresponding author on reasonable request.

## Endnotes

^1^
The online questionnaire is provided as supplementary material.

^2^
The interview guides are provided as supplementary material.

^3^
The category systems with the main and sub‐categories that were developed are provided as supplementary material.

^4^
It is specified whether the results discussed in this section are derived from the interviews or the online questionnaire.
