# Supplementary material for: Professional Digital Counselling for Eating Disorders in Germany: Results of the DigiBEssst Project Survey on the Perspectives and Experiences of Health Professionals, Individuals With Eating Disorders, and Carers
Source: Eur Eat Disord Rev. 2024 Dec 19;33(3):562–74. doi: 10.1002/erv.3164 (PMC11965544; doi:10.1002/erv.3164)
Supplement: Supplementary file 1 — Supporting Information S1 [file ERV-33-562-s004.docx]

## Category systems for the interviews with professionals, individuals with eating disorders, and carers

### Category system: Interviews with professionals

1. Sociodemographic characteristics of the interviewee
 1.1 Age
 1.2 Gender
 1.3 Education and qualification
 1.4 Employment in the field of eating disorders
 1.5 Employment and training in online counseling
2. Practical contact with online counseling
 2.1 Duration and frequency
 2.2 Setting and target group
 2.3 Media usage
 2.4 Impact of the COVID-19 pandemic
3. Understanding of the term "online counseling"
4. Access and availability of online counseling
 4.1 Opportunity of online counseling: flexibility for and accessibility to individuals with eating disorders
 4.2 Findability and design of access to online counseling
 4.3 Participatory selection of counseling format
 4.4 Access via the medium "messenger"
5. Diversity in online counseling
 5.1 Age
 5.2 Gender and gender identity
 5.3 Language and culture
 5.4 Comorbidities and other challenges
 5.5 Individuals with disabilities and digital accessibility
 5.6 Other aspects of diversity
6. Specifics regarding the type of eating disorder
 6.1 Assessment of the type of eating disorder before or during online counseling
 6.2 Specifics of different types of eating disorders in online counseling
7. Blended (online) counseling
8. Qualification and competencies of online counselors
 8.1 Qualifications and further training
 8.2 Basic knowledge and attitudes in counseling
 8.3 Technical competencies
 8.4 Specialized knowledge related to eating disorders
 8.5 Reading and writing skills
 8.6 Competencies for video-based counseling
9. Relationship building
 9.1 Relationship building across counseling formats
 9.2 Relationship building in text-based online counseling
 9.3 Relationship building in video-based online counseling
 9.4 Opportunity of online counseling: greater openness and reduced shame in online counseling
 9.5 Challenge of online counseling: absence of physical presence
10. Difficult situations and boundaries
 10.1 Boundaries of online counseling for eating disorders
 10.2 Challenges in assessing boundaries
 10.3 Crisis situations
11. Self-care for online counselors
12. Online counseling in multi-person settings
 12.1 Online counseling with relatives
 12.2 Online counseling with groups
13. Online counselors’ attitude towards online counseling
14. Concept for online counseling
15. Financial aspects
16. Personnel and time resources
 16.1 Personnel resources
 16.2 Time resources
17. Team collaboration and external cooperation
 17.1 Collaboration within the team
 17.2 Networking and referrals
18. Technical equipment
19. Legal frameworks
20. Quality management
21. Feedback on the project and the development of quality guidelines

### Category system: Interviews with individuals with eating disorders

1. Sociodemographic characteristics of the interviewee
 1.1 Age
 1.2 Gender
 1.3 Type of eating disorder
2. Motivation for participation
3. Practical contact with online counseling
 3.1 Duration and frequency
 3.2 Setting
 3.3 Media usage
 3.4 Impact of the COVID-19 pandemic
4. Understanding of the term "online counseling"
5. Access and availability of online counseling
 5.1 Opportunity of online counseling: flexibility for and accessibility to individuals with eating disorders
 5.2 Findability and design of access to online counseling
 5.3 Participatory selection of counseling format
 5.4 Access via the medium "messenger" and social media
6. Diversity in online counseling
 6.1 Age
 6.2 Gender and gender identity
 6.3 Language and culture
 6.4 Comorbidities and other challenges
7. Specifics regarding the type of eating disorder
8. Blended (online) counseling
9. Qualification and competencies of online counselors
 9.1 Qualification, further training, and basic competencies
 9.2 Specialized competencies related to eating disorders
 9.3 Providing clear framework and structure
10. Relationship building
 10.1 Relationship building
 10.2 Opportunity of online counseling: greater openness and reduced shame
 10.3 Challenge of online counseling: absence of physical presence
11. Difficult situations and boundaries
 11.1 Boundaries of online counseling for eating disorders
 11.2 Challenges in assessing boundaries
 11.3 Challenges in text-based counseling
 11.4 Challenges in video-based counseling
 11.5 Challenge of online counseling: counseling in one's own home
12. Online counseling in multi-person settings
 12.1 Online counseling with relatives
 12.2 Online groups for affected individuals
13. Financial aspects
14. Networking and referrals
15. Technology
16. Legal frameworks
17. Evaluation of online counseling
18. Feedback on the project and the development of quality guidelines

### Category system: Interviews with carers

1. Sociodemographic characteristics of the interviewee
 1.1 Role of relatives
 1.2 Age
 1.3 Gender
2. Motivation for participation
3. Practical contact with online counseling
 3.1 Duration and frequency
 3.2 Setting
 3.3 Media usage
 3.4 Impact of the COVID-19 pandemic
4. Understanding of the term "online counseling"
5. Access and availability of online counseling
 5.1 Opportunity of online counseling: Temporal and spatial flexibility
 5.2 Findability and design of access to online counseling
 5.3 Forms or questionnaires before online counseling
 5.4 Participation and needs-based services
 5.5 Blended counseling
 5.6 Access via the medium "messenger"
6. Diversity in online counseling
 6.1 Role of relatives
 6.2 Physical and psychological challenges and illnesses
 6.3 Language and culture
 6.4 Age
 6.5 Gender
 6.6 Other diversity aspects
7. Content related to the loved ones affected by eating disorders
8. Qualification and competencies of online counselors
 8.1 Basic knowledge and attitudes
 8.2 Providing clear agreements, structure, and regulations
 8.3 Dealing with written communication
 8.4 Application of methods and tools
 8.5 Expertise in the field of eating disorders
9. Relationship and trust
 9.1 Relationship building
 9.3 Absence of physical presence
10. Boundaries of online counseling
 10.1 Boundaries in online counseling
 10.2 Counseling in one's own home
11. Handling crisis situations
12. Online counseling in multi-person settings
 12.1 Online counseling with family and couples
 12.2 Online counseling with groups
13. Financial, personnel, and time aspects
14. Networking and referrals
15. Technology
 15.1 Technical aspects and competencies of professionals
 15.2 Technical competencies of clients
16. Legal frameworks
17. Quality management, evaluation, and feedback
18. Experiences with unprofessional online counseling
19. Feedback on the project and the development of quality guidelines
