# Supplementary material for: Professional Digital Counselling for Eating Disorders in Germany: Results of the DigiBEssst Project Survey on the Perspectives and Experiences of Health Professionals, Individuals With Eating Disorders, and Carers
Source: Eur Eat Disord Rev. 2024 Dec 19;33(3):562–74. doi: 10.1002/erv.3164 (PMC11965544; doi:10.1002/erv.3164)
Supplement: Supplementary file 4 — Supporting Information S4 [file ERV-33-562-s005.docx]

## Main questions from the interview guide for carers^[[1]](#footnote-1)^

### Information Phase

- Greeting and thanks for participation
- Handling the camera during the interview
- Introduction of the interviewer
- Information about the research project
- Explanation of the interview process
- Activation of audio recording and re-obtaining verbal consent for this
- Information on data protection and obtaining informed consent

### Introduction Phase

1. Why did you offer to participate in an interview?
2. What do you understand by "online counseling"?
3. **Main Phase**

***Topic Block: Experiences with Online Counseling for Eating Disorders***

1. What points of contact with online counseling for eating disorders have you had as a carer?
2. For what reasons did you, as a carer, choose *online* counseling?
3. What, in your opinion as a carer, speaks against using *online* counseling?
4. From your perspective, what specific considerations should be taken into account in online counseling for family members (on the topic of eating disorders)?
5. Via which medium or media (e.g., email, video, chat, messenger, forum, other) did you use online counseling for eating disorders and for what reasons?
6. To what extent have you used online counseling for eating disorders alone or together with other people (e.g., family members, individual with eating disorder)?

***Topic Block: Standards and Qualifications for Online Counseling in Eating Disorders***

1. How do you recognize a professional online counseling service for family members of individuals with eating disorders?
2. What helped you, as a carer, the most in online counseling for eating disorders?
3. In your view, what is necessary to further improve the online counseling service for family members of individuals with eating disorders?

***Topic Block: Diversity in Online Counseling for Eating Disorders***

1. To what extent was your specific role as a family member (as father/mother/partner) relevant?
2. To what extent did aspects such as age, language, or your own mental or physical illnesses/conditions play a role for you in online counseling?
3. To what extent did your gender play a role in online counseling for eating disorders?

***Topic Block: Concluding and Demographic Questions***

1. Do you have any suggestions, wishes, or ideas for the development of quality guidelines in the project?
2. Which federal state are you from, and do you live in a rural area or in a city (small town, large city)?
3. How old are you?
4. **Closing Phase**
5. Would you like to add anything else or do you have any feedback?
6. How do you feel leaving this conversation, and do you need anything from me to ensure you leave feeling well?

1. Each group (professionals, individuals with eating disorders, carers) had slightly different interview guides. This is the interview guide for carers of individuals with eating disorders. The original interview guide is further divided into sub-questions. This document contains the main questions. [↑](#footnote-ref-1)
