# Supplementary material for: Professional Digital Counselling for Eating Disorders in Germany: Results of the DigiBEssst Project Survey on the Perspectives and Experiences of Health Professionals, Individuals With Eating Disorders, and Carers
Source: Eur Eat Disord Rev. 2024 Dec 19;33(3):562–74. doi: 10.1002/erv.3164 (PMC11965544; doi:10.1002/erv.3164)
Supplement: Supplementary file 6 — Supporting Information S6 [file ERV-33-562-s003.docx]

## Main questions from the interview guide for professionals at eating disorder counseling centers^[[1]](#footnote-1)^

### Information Phase

- Greeting and thanks for participation
- Handling the camera during the interview
- Introduction of the interviewer
- Information about the research project
- Explanation of the interview process
- Activation of audio recording and re-obtaining verbal consent for this
- Information on data protection and obtaining informed consent

### Introduction Phase

1. What is your job title?
2. What do you understand by "online counseling"?
3. What interactions have you had with online counseling for eating disorders?
4. **Main Phase**

***Topic Block: Consideration of Specifics in Eating Disorders***

1. What specific aspects need to be considered in online counseling for individuals with eating disorders compared to other client groups, and how do you handle these aspects?
2. When is online counseling suitable for people with eating disorders (indication), and when is it not suitable (contraindication)?
3. How do differences in the type of eating disorder (e.g., anorexia nervosa, bulimia nervosa, binge eating disorder) affect online counseling?
4. If you offer online *and* face-to-face consultations at your counseling center: How do clients of the online counseling service for eating disorders differ from those of the face-to-face counseling service?

***Topic Block: Diversity in Online Counseling for Eating Disorders***

1. To what extent does gender play a role in online counseling for eating disorders?
2. How does the diversity of clients affect your online counseling service for eating disorders (e.g., age, language, mental or physical illnesses)?

***Topic Block: Standards and Qualifications for Online Counseling in Eating Disorders***

1. What, in your opinion, constitutes a professional online counseling service for eating disorders?
2. What is important in online counseling for eating disorders regarding competence in methods or counseling and relational skills?
3. What is important in managing crisis situations and difficult communication scenarios in online counseling for eating disorders?
4. What is important regarding (self-)reflection and self-care in online counseling for eating disorders?
5. What is essential for teamwork and external collaboration in online counseling for eating disorders?
6. What are the financial and personnel support and resources needed for online counseling in eating disorders?
7. What is crucial for quality management in online counseling for eating disorders?
8. What are the necessary technical resources and competencies for online counseling in eating disorders?

***Topic Block: Media in Online Counseling for Eating Disorders***

1. What experiences do you have with blended counseling?
2. What experiences do you have with online counseling via messenger services?

***Topic Block: Concluding and Demographic Questions***

1. Do you have any suggestions, wishes, or ideas for the development of quality guidelines in the project?
2. How old are you?
3. What gender do you identify with?
4. **Closing Phase**
5. Would you like to add anything else or do you have any feedback?
6. How do you feel leaving this conversation, and do you need anything from me to ensure you leave feeling satisfied?

1. Each group (professionals, individuals with eating disorders, carers) had slightly different interview guides. This is the interview guide for professionals with experience in online counseling for eating disorders. The original interview guide is further divided into sub-questions. This document contains the main questions. [↑](#footnote-ref-1)
