# Supplementary material for: Professional Digital Counselling for Eating Disorders in Germany: Results of the DigiBEssst Project Survey on the Perspectives and Experiences of Health Professionals, Individuals With Eating Disorders, and Carers
Source: Eur Eat Disord Rev. 2024 Dec 19;33(3):562–74. doi: 10.1002/erv.3164 (PMC11965544; doi:10.1002/erv.3164)
Supplement: Supplementary file 7 — Supporting Information S7 [file ERV-33-562-s007.docx]

**Online Questionnaire – 03.05.2022**

Hofer, Anna*; Borse, Sigrid; Hasenöhrl, Cäcilia; Harrach, Kathrin; Ehrlich, Stefan; Schnebel, Andreas; Wunderer, Eva

Preliminary Note: Please note that the questionnaire was originally administered in German, and the English translation would need to be tested and validated.

**Page 1**

Thank you for participating in the survey “Digital counseling in professional counseling centers for eating disorders 2022”

Please answer the questionnaire from the perspective of your counseling center.

If you need to interrupt the questionnaire, for example, to gather more information or due to accidentally closing the browser window, the questionnaire can be resumed through the link sent to you by email and continued from where you left off.

It is important to consider that the page of the questionnaire is only saved after clicking the "Next" button. Only click "Next" and close the browser after the page is saved.

Please use the "Next" and "Back" buttons at the bottom of the page to navigate within the questionnaire. Do not use the browser’s "Back" button.

Please remember to complete the questionnaire, as your answers cannot be included in the evaluation unless you finish it.

Your data will be kept confidential, and it will not be possible to trace the information back to the individual. For details about data protection, you can also refer to the study information previously sent to you via email (link to download).

Before you proceed to the actual survey, we need your explicit consent:

☐ I hereby consent to the processing of my data in accordance with the provided information. I can withdraw my consent at any time. The withdrawal does not affect the lawfulness of the data processing based on the consent given until the withdrawal.

The following questions can only be answered meaningfully if your counseling center offers online counseling for eating disorders:

☐ We are a professional counseling center that offers online counseling for eating disorders.

For any further questions, you can contact the lead research Anna Hofer (anna.hofer@haw-landshut.de). For other questions, you can also contact Eva Wunderer (eva.wunderer@haw-landshut.de), or the project management at the BFE (bfe-essstoerungen@gmx.de).

Interrupt Questionnaire | Next

Implementation of the online questionnaire and survey conduct: Julia Post – Applied Social Research

**Page 2**

Before we begin, here is a brief explanation of some of the terms we will use in the survey:

Affected individuals: People with eating disorders

Family members: Family members of people with eating disorders

Individuals seeking help: People with eating disorders, their family members, and individuals from their social circle (e.g., friends) who contact the counseling center

Online counselors: All professionals who offer online counseling within your counseling center

We kindly ask you to answer all the following questions. Every answer and all your experiences with online counseling in the field of eating disorders are crucial for us in gathering important information for the development of quality guidelines. For this reason, some questions are marked as mandatory, which you must answer to proceed to the next page of the survey. If you overlook any questions, you will receive a reminder in red. Thank you very much!

Interrupt Survey | Next

**Page 3**

**Online Counseling Services at Your Counseling Center**

**1. Key Information About the Counseling Center**
At the beginning, we will ask a few key details about your counseling center.

1.1 In which federal state is your counseling center located?
- Baden-Württemberg
- Bavaria
- Berlin
- Brandenburg
- Bremen
- Hamburg
- Hesse
- Mecklenburg-Western Pomerania
- Lower Saxony
- North Rhine-Westphalia
- Rhineland-Palatinate
- Saarland
- Saxony
- Saxony-Anhalt
- Schleswig-Holstein
- Thuringia

1.2 How large is the community or city in which your counseling center is located?
- Rural area (less than 5,000 inhabitants)
- Small town (5,000 to less than 20,000 inhabitants)
- Medium-sized town (20,000 to less than 100,000 inhabitants)
- Large city (more than 100,000 inhabitants)

Back | Interrupt Survey | Next

**Page 4**

**2. Online Counseling Services in the Field of Eating Disorders**
This section is specifically about the online counseling services for eating disorders offered by your counseling center.

2.1 How is your online counseling accessible?
Please select all that apply.
- Website of the counseling center
- Specific counseling portal (e.g., B. Beranet, AYGOnet)
- Email of the counseling center
- Social media
- Other: ____________________

2.2 Which media are you currently using for online counseling?
Please select all that apply.
- Email
- Chat
- Forum
- Messenger
- Video
- Other: ____________________

2.3 Which media would you like to continue using or newly adopt for online counseling in the future?
Please select all that apply.
- Email
- Chat
- Forum
- Messenger
- Video
- Other: ____________________

2.4 Does your counseling center also offer pure face-to-face counseling, meaning counseling exclusively in person?
- Yes
- No

2.5 Does your counseling center also offer telephone counseling alongside online counseling?
- Yes
- No

2.6 Does your counseling center offer blended counseling, combining online and in-person counseling for a person seeking help?
- Yes
- No
- Other: ____________________

2.7 For which applications do you use online services?
Please select all that apply.
- Primary/universal prevention (this means preventive services for everyone, even those without eating disorders, e.g., in schools)
- Counseling in terms of clearing (diagnostic clarification + referral + motivation)
- Counseling as a way to bridge waiting times (e.g., waiting for a therapy place)
- Counseling as aftercare
- Further education
- Specialist counseling
- Other: ____________________

2.8 In which setting and for which target group does online counseling take place at your counseling center?
Please select all that apply.
- Individual counseling for affected individuals
- Group counseling for affected individuals
- Couples counseling (simultaneously for affected individuals and their partners)
- Family counseling (simultaneously for affected individuals and their family members)
- Counseling for affected individuals with other people from their social environment (simultaneously, e.g., friends, teachers, etc.)
- Counseling for family members (without the affected individuals)
- Counseling for people in the social environment of affected individuals, such as friends, teachers (without the affected individuals)
- Counseling for professionals
- Other: ____________________

2.9 In which setting and for which target group does online counseling take place most frequently at your counseling center?
Please sort the options you selected in question 2.8 based on frequency. Drag the cards from right to left in the boxes.
- Individual counseling for affected individuals
- Group counseling for affected individuals
- Couples counseling (affected individuals and partner)
- Counseling for family members (without the affected individuals)
- Family counseling for affected individuals and family members
- Counseling for people in the social environment of affected individuals (e.g., friends, teachers without the affected individuals)
- Counseling for professionals
- Other as indicated in 2.8

2.10 What percentage of counseling for individuals with eating disorders takes place online at your counseling center? _______ %

2.11 What percentage of counseling for family members takes place online at your counseling center? _______ %

2.12 Who offers online counseling at your counseling center?
Please select all that apply.
- Social educators/social workers
- Educators
- Psychologists
- Licensed psychotherapists
- Doctors
- Nutritionists (e.g., dietitians)
- Other: ____________________

2.13 In which languages is online counseling offered at your counseling center?
Please select all that apply.
- German
- English
- Spanish
- French
- Italian
- Turkish
- Greek
- Russian
- Polish
- Kurdish
- Arabic
- Serbian
- Croatian
- Sign language
- Other: ____________________

2.14 In which languages do you inform about your online counseling services on your website?
Please select all that apply.
- German
- English
- Spanish
- French
- Italian
- Turkish
- Greek
- Russian
- Polish
- Kurdish
- Arabic
- Serbian
- Croatian
- Sign language
- Other: ____________________

Back | Interrupt Survey | Next

**Page 5**

**Experiences with Online Counseling for Eating Disorders**

**3. Changes Due to the COVID-19 Pandemic**
We are now interested in how the COVID-19 pandemic has affected online counseling in your counseling center.

3.1 Has online counseling already been offered at your counseling center before the COVID-19 pandemic?
- Yes
- No

3.2 How has usage of online counseling changed since the beginning of the COVID-19 pandemic in your counseling center?
Individuals with eating disorders ...
- ... use online counseling more.
- ... use online counseling less.
- ... use online counseling the same as before the pandemic.

Family members ...
- ... use online counseling more.
- ... use online counseling less.
- ... use online counseling the same as before the pandemic.

The professionals at your counseling center ...
- ... use online counseling more.
- ... use online counseling less.
- ... use online counseling the same as before the pandemic.

3.3 To what extent has the perspective or attitude towards online counseling changed among the professionals at your counseling center due to the COVID-19 pandemic?
Please rate the following aspects:

| Online counseling is now seen as a good alternative to in-person counseling by most professionals at the counseling center. | Does not apply at all      applies completely |
| --- | --- |
| The advantages of online counseling are now more recognized by professionals. | Does not apply at all      applies completely |
| The disadvantages of online counseling are now more recognized by professionals. | Does not apply at all      applies completely |
| For most professionals, online counseling is still primarily seen as an emergency solution during the COVID-19 pandemic. | Does not apply at all      applies completely |
| Other: _____________________________ | |

3.4 How do you expect the usage of online counseling to develop at your counseling center after the end of the COVID-19 pandemic compared with during the pandemic?
Individuals with eating disorders ...
- ... will use online counseling more in the future than during the pandemic.
- ... will use online counseling less in the future than during the pandemic.
- ... will use online counseling the same as during the pandemic.

Family members ...
- ... will use online counseling more in the future than during the pandemic.
- ... will use online counseling less in the future than during the pandemic.
- ... will use online counseling the same as during the pandemic.

The professionals at your counseling center ...
- ... will use online counseling more in the future than during the pandemic.
- ... will use online counseling less in the future than during the pandemic.
- ... will use online counseling the same as during the pandemic.

Back | Interrupt Survey | Next

**Page 6**
**4. Standards for Online Counseling in Eating Disorders**
In this section of the online survey, the focus is on the standards of online counseling in your counseling center. Below you will find questions on various topics and aspects, which you will assess using scales, and you may also add your aspects.

4.1 Does your counseling center have a concept with standards for online counseling that you follow as a professional?
Please select all that apply.
- Yes, we have developed our own concept for online counseling in our counseling center. We have based it on: ____________________
- Yes, we follow general standards for online counseling, namely: ____________________
- We largely adopt our concept for in-person counseling for online counseling.
- No
- Other: ____________________

4.2 Below, we have compiled a number of aspects related to the **institutional quality assurance of online counseling**. How do you assess the relevance of the individual aspects specifically for online counseling, and how well are they implemented in your counseling center?
If, for example, your online counseling service is still in the development phase, you can choose “Other” on the implementation scale below and give us a brief explanation (e.g., online counseling is still being established, so no specific concept exists yet).

|  | How relevant is this aspect? | How well is this aspect implemented in your counseling center? |
| --- | --- | --- |
| **Specific concept** for **online counseling** | not important at all      very important | very poor      very good  Other: ____________________ |
| **Financial support** for **online counseling** | not important at all      very important | very poor      very good  Other: ____________________ |
| **Technical equipment** for **online counseling** | not important at all      very important | very poor      very good  Other: ____________________ |
| **Staff resources** for **online counseling, administration and technical support for online counseling** | not important at all      very important | very poor      very good  Other: ____________________ |
| **Home office with adequate equipment** for **online counseling** | not important at all      very important | very poor      very good  Other: ____________________ |
| **System stability** in **online counseling** (e.g., minimizing the risk of connection loss due to technical problems) | not important at all      very important | very poor      very good  Other: ____________________ |
| **Defined timeframe for expecting responses in online counseling** | not important at all      very important | very poor      very good  Other: ____________________ |
| **Defined time budget for online counseling (e.g., time per client, determination of the number of contacts)** | not important at all      very important | very poor      very good  Other: ____________________ |
| **Defined procedures in crisis situations** in **online counseling** (e.g., suicidality in individuals seeking help) | not important at all      very important | very poor      very good  Other: ____________________ |
| **Coordination of organizational processes** regarding **online counseling** (e.g., regulations regarding the responsibility of individual cases, handovers, case take overs, and continuation of counseling requests) | not important at all      very important | very poor      very good  Other: ____________________ |
| **Multi-professionalism** in **online counseling** **(involvement of different professions, depending on the specific needs of the clients)** | not important at all      very important | very poor      very good  Other: ____________________ |
| **Intervision, team meetings, case discussions specific to online counseling** | not important at all      very important | very poor      very good  Other: ____________________ |
| **External supervision** specific to **online counseling** | not important at all      very important | very poor      very good  Other: ____________________ |
| **Networking and referral of online counseling clients to appropriate facilities** | not important at all      very important | very poor      very good  Other: ____________________ |
| **Regulations for documentation** of **online counseling** (e.g., storage of online counseling session data) | not important at all      very important | very poor      very good  Other: ____________________ |
| **Evaluation** of **online counseling** (e.g., satisfaction of clients) | not important at all      very important | very poor      very good  Other: ____________________ |
| **Complaints management** for **online counseling clients** | not important at all      very important | very poor      very good  Other: ____________________ |
| **Specific qualifications for professionals providing online counseling in the counseling center (e.g., further training)** | not important at all      very important | very poor      very good  Other: ____________________ |

Please provide an explanation here if you have selected “Other” somewhere: ____________________

4.3 Do you have any additions or comments on the aspects mentioned in 4.2? If so, please write them here.
Please use one field per comment.

____________________

4.4 In your view, what would be desirable to better implement the aspects mentioned in 4.2?
Please use one field per comment.

____________________

Back | Interrupt Survey | Next

**Page 7**

**5. Qualifications for Online Counseling**

5.1 Below, we have listed a number of specific competencies and knowledge areas for online counseling. How do you assess the relevance of each aspect, and how well are these skills present among online counselors in your counseling center?
If, for example, your online counseling service is still being set up, you can select "Other" on the implementation scale below and give us a brief explanation (e.g., online counseling is still being developed, so no specific methodological competence is yet available for online counseling).

|  | How relevant is this aspect? | How well is this aspect implemented in your counseling center? |
| --- | --- | --- |
| **Technical competence** for **online counseling** (e.g., computer skills) | not important at all      very important | very poor      very good  Other: ____________________ |
| **Specific reading, writing, and text competence** for **online counseling** **(e.g., reading between the lines and responding, using emoticons and abbreviations, fast typing)** | not important at all      very important | very poor      very good  Other: ____________________ |
| **Specific methodological competence** for **online counseling** **(e.g., using tools such as online whiteboard, adapting methods to online counseling)** | not important at all      very important | very poor      very good  Other: ____________________ |
| **Knowledge of legal frameworks** for **online counseling** | not important at all      very important | very poor      very good  Other: ____________________ |
| **Establishment of professional working relationships in online counseling (e.g., facilitating a helping process despite anonymity, handling lower commitment in online counseling)** | not important at all      very important | very poor      very good  Other: ____________________ |
| **Assessment of indication and contraindication of online counseling for people with eating disorders** | not important at all      very important | very poor      very good  Other: ____________________ |
| **Using different forms of online counseling with their respective special features appropriately (e.g., email, video, chat)** | not important at all      very important | very poor      very good  Other: ____________________ |
| **Crisis intervention and emergency planning** in **online counseling** | not important at all      very important | very poor      very good  Other: ____________________ |
| **Dealing with difficult communication situations** in **online counseling (e.g., prolific writers, communication breakdowns)** | not important at all      very important | very poor      very good  Other: ____________________ |
| **Establishing a reflective open attitude toward online counseling** | not important at all      very important | very poor      very good  Other: ____________________ |
| **Consideration of self-care in online counseling (e.g., setting boundaries)** | not important at all      very important | very poor      very good  Other: ____________________ |

Please provide an explanation here if you selected “Other”: ____________________

5.2 Do you have any additions or comments on the aspects mentioned in 5.1? If so, please write them here.
Please use one field per comment.
____________________

5.3 In your view, what would be desirable to better implement the aspects mentioned in 5.1?
Please use one field per comment.

____________________

Back | Interrupt Survey | Next

**Page 8**

**6. Specific Aspects of Online Counseling for People with Eating Disorders**

6.1 What specific aspects should be considered in online counseling for individuals with eating disorders? Please provide an assessment of the listed aspects.
If only video counseling is offered at your center, you can select "Cannot assess" for text-based online counseling and provide us with a brief explanation below.

| For individuals with eating disorders, **confronting their own image during video counseling** is a high burden | does not apply at all      applies completely  cannot assess |
| --- | --- |
| The **weight** of individuals with eating disorders may be less accurately assessed in **text-based online counseling** than in in-person counseling | does not apply at all      applies completely  cannot assess |
| The **weight** of individuals with eating disorders may be less accurately assessed in **video counseling** than in in-person counseling | does not apply at all      applies completely  cannot assess |
| **Potential physical endangerment** may be less accurately recognized in **text-based online counseling** compared with in-person counseling | does not apply at all      applies completely  cannot assess |
| **Potential physical endangerment** may be less accurately recognized in **video counseling** compared with in-person counseling | does not apply at all      applies completely  cannot assess |
| **Indications of extreme vomiting** may be less effectively recognized in **text-based online counseling** compared with in-person counseling | does not apply at all      applies completely  cannot assess |
| **Indications of severe vomiting** may be less effectively discerned in **video counseling** compared with in-person counseling | does not apply at all      applies completely  cannot assess |
| In **anonymous text-based online counseling**, individuals with eating disorders experience **less shame** than in other forms of counseling | does not apply at all      applies completely  cannot assess |
| Anonymous text-based online counseling **reduces the inhibition for male individuals** to seek help | does not apply at all      applies completely  cannot assess |
| Anonymous text-based online counseling **reduces the inhibition for female individuals** to seek help | does not apply at all      applies completely  cannot assess |
| Anonymous text-based online counseling **reduces the inhibition for gender-diverse individuals** to seek help | does not apply at all      applies completely  cannot assess |
| In text-based online counseling, individuals with eating disorders experience **more control and autonomy** than in other forms of counseling | does not apply at all      applies completely  cannot assess |
| Other: ____________________ | |

Please provide a brief explanation here if you selected “Cannot assess” somewhere: ____________________

6.2 Please add any further specific aspects that should be considered in online counseling for individuals with eating disorders, and/or provide comments on the aspects mentioned in 6.1.
Please use one field per comment.

____________________

6.3 What criteria do you use to determine whether online counseling is suitable for individuals with eating disorders? Are there specific exclusion criteria, and how are cases handled when online counseling is considered 'unsuitable' for the individual?
Please use one field per comment.

____________________

Back | Interrupt Survey | Next

**Page 9**

**7. Final Question**We would be happy if you could answer our final question:

7.1 What do you expect from the quality guidelines to be developed within the framework of the DigiBEssst project? Please use one field per comment.

____________________

7.2 Here you have space for additional ideas, comments, and suggestions regarding the online survey. Please use one field per comment.

____________________

**Please note:** You are on the last page of the questionnaire. By clicking on “Submit,” the data you have entered will be submitted — reopening the questionnaire will not be possible.

To submit the questionnaire, please click on the “Submit” button at the bottom right.

Back | Interrupt Survey | Submit

**Page 10**

**Thank you very much for your participation in the online survey for the DigiBEssst project!**

Your expertise is the foundation of the quality guidelines, which will then be returned to you and benefit practical work. We will be happy to inform you about our results.

To gain even deeper insights into your expertise and experiences regarding online counseling in the field of eating disorder support, we warmly invite you to participate in a **follow-up interview** in autumn/winter 2022 and would be very pleased if you could take part. If you are interested in an interview, feel free to send us an **email** (email: anna.hofer@haw-landshut.de). This way, we can provide you with further information about the planned interview. Thank you!

Your responses have been saved. You can now close the browser window.
